# New Era for Usage of Serum Liver Enzymes as A Promising Horizon for the Prediction of Non-Alcoholic Fatty Liver Disease

**DOI:** 10.3889/oamjms.2016.092

**Published:** 2016-09-01

**Authors:** Ahmed Abd Allah Salman, Soheir Abd Elfattah Aboelfadl, Mona Abd Elmenem Heagzy

**Affiliations:** *Cairo University, Faculty of Medicine - Internal Medicine, Cairo, Egypt*

**Keywords:** Liver, enzymes, NASH, prediction, Horizon

## Abstract

**BACKGROUND:**

Liver histology remains the gold standard for assessing non-alcoholic fatty liver disease (NAFLD). Noninvasive serological markers and radiological methods have been developed to evaluate steatosis to avoid biopsy.

**AIM:**

To put cutoff value for liver enzymes that could predict non-alcoholic steatohepatitis (NASH).

**PATIENTS AND METHODS:**

This study was conducted on 54 patients (with NAFLD diagnosed by the US). Patients were subjected to history, physical, anthropometric measurements, investigations including liver enzymes, abdominal US, and liver biopsy. According to biopsy results, patients were subdivided according to NASH development. Also, biopsy results were correlated to the levels of liver enzymes.

**RESULTS:**

Forty-seven patients who were suspected to have NAFLD by sonar were confirmed by biopsy. There was a significant correlation between steatosis degree in biopsy and sonar. Correlation study between steatosis in biopsy and ALT level showed highly significant positive correlation. Correlation study between steatosis in biopsy on one side & AST and GGT on the other side showed significant positive correlation. Cutoff value for detection of NASH using ALT & AST & and GGT were 50.5, 56, 60.5 respectively with sensitivity = 95.5, 90.5, 86.4 % and specificity = 93.8, 100, 87.5%.

**CONCLUSION:**

Cut off values of liver enzymes can be combined with abdominal sonar to predict NASH.

## Introduction

Non-alcoholic fatty liver disease (NAFLD) is one of the manifestations of metabolic syndrome, that include hypertension, diabetes, excess body fat around the waist or abnormal cholesterol levels that can predispose to heart disease, diabetes, and stroke [1].

The prevalence of NAFLD is very high in obese and overweight individuals. NAFLD spectrum ranges from simple fatty infiltration to more severe manifestations such as hepatocyte inflammation, hepatic fibrosis, and cirrhosis [2].

Liver biopsy is considered the best method for determining disease stage in NAFLD. A biopsy is not convenient for very large studies and especially for studying hepatic fibrosis progression as it is invasive. Moreover, histological assessment of NAFLD may cause sampling error and can result in misinterpretation of the fibrosis score especially in cases of insufficient specimen [3].

Ultrasound (US) is now the most widely used investigation for screening asymptomatic people with abnormal liver functions and suspected fatty liver [4]. Noninvasive serological markers may be used to assess steatosis and inflammation to avoid the need for biopsy [5]. In patients with NAFLD, serum Gamma-glutamyltransferase (GGT) is often raised and it has been linked to increased death rate [4]. Alanine aminotransferase (ALT) can be used as an indicator for liver injury. It is an important issue to differentiate simple steatosis from steatohepatitis because steatohepatitis has a progressive course and can cause liver failure [6].

The aim of this study was to evaluate liver enzymes and ultrasonic staging of hepatic steatosis as predictors for the severity of NAFLD, and trying to put cutoff values for liver enzymes that could predict the development of non-alcoholic steatohepatitis (NASH).

## Patients and Methods

This study was performed in Kasr El-Ainy hospital, Internal Medicine outpatient clinic over a period of 11 months on 54 obese subjects (BMI “body mass index” < 30 kg/m^2). They came complaining of dyspepsia, 17 patients (31.5 %), osteoarthritis, 15 patients (27.8 %), back pain, 12 patients (22.2 %) and obesity, 10 patients (18.5 %).

The selection of participants in this study was based on the following Inclusion criteria:

1. All participants aged above 18 years.
2. BMI above 30 kg/m^2.
3. All patients showed a picture of bright liver ± hepatomegaly on abdominal ultrasound.

Exclusion criteria:

1. Patients are known to be alcoholic.
2. Patients are known to be positive for hepatitis C or B.
3. Patients are known to be diabetic.

Each patient was subjected to full medical history, full clinical examination, and anthropometric measurements (weight and height of each participant were measured while the participant was clothed only in a light gown, and the BMI was calculated as body weight in kilogram (kg) divided by height squared in meters “m” (Kg/m^2), also waist circumference `was measured midway between rib margin and the iliac crest in a standing position by the same examiner.

Serum biochemistry profile (including serum levels of ALT, AST “aspartate transferase” and GGT) was done for every participant.

Abdominal US was performed by the same operator using a Toshiba Apilo xv scanner equipped with a broadband 5.3 megahertz curved array probe to assess the presence of liver steatosis (bright liver). Liver biopsy was taken for all patients who were suspected to have NAFLD by the abdominal US. Liver biopsy was fixed in ten percent neutral buffered formalin then embedded in paraffin blocks. Five micrometre thick sections were cut and stained with hematoxylin and eosin and examined under light microscope for histopathological diagnosis and scoring using NAS “NAFLD activity score” scoring system according to Histological Scoring System for Nonalcoholic Fatty Liver Disease [7]. This scoring system addresses the full spectrum of lesions of NAFLD and allows a diagnostic categorization into NASH, borderline NASH or not NASH. Fibrosis staging was evaluated (separately from NASH) from 0 to 4 scales [8]. As regarding statistical analysis, we considered borderline NASH and NASH as one group to facilitate comparison between the 2 studied groups (NASH and non-NASH).

### Ethics

The study protocol conformed to ethical guidelines of the 1975 declaration of Helsinki, approved by Cairo University Research Ethics Committee (REC) (No.n-7-2015 in 28-5-2015).

A written informed consent was obtained from all patients participating in the study. Data were statistically described in terms of mean ± standard deviation (± SD), median and range, or frequencies (number of cases) and percentages when appropriate. Comparison of numerical variables between the study groups was done using Kruskal-Wallis test with posthoc multiple 2-group comparisons. For comparing categorical data, Chi-square (χ^2^) test was performed. The exact test was used instead when the expected frequency is less than 5. Agreement between ultrasound and biopsy results was tested using kappa statistic. P values less than 0.05 was considered statistically significant. All statistical calculations were done using computer programs SPSS (Statistical Package for the Social Science; SPSS Inc., Chicago, IL, USA) version 15 for Microsoft Windows.

Receiver operator characteristic (ROC) curves were derived and area under the curve (AUC) analysis performed to get the best cutoff values for predicting NASH.

## Results

The present study included 54 patients, suspected to have NAFLD (clinically & by abdominal ultrasound); and selected from the outpatient clinic. There were 50 females (92.6%) and 4 males (7.4%). The age of the participants ranged from 18- 60 years with a mean ± SD = 43.17 ± 7.371. BMI of the participants ranged from 30.2kg/m ^2 to 42.1 kg/m^2 with a mean ± SD = 34.774 ± 3.7613. Abdominal US showed a high sensitivity in detection of NAFLD as 87% of the patients who were suspected to have NAFLD by sonar were confirmed to have NAFLD by liver biopsy (47 patients out of 54 patients). To facilitate statistical data, we considered fibrofatty and grade 1 steatosis in sonar as one group (group 1), also we considered grade 3 and 4 steatosis as one group (group 3). As to liver biopsy, we considered grade 0 and 1 steatosis as one group (group 1), then we correlate grading of steatosis by the abdominal US to grading of steatosis by liver biopsy.

**Figure 1:**
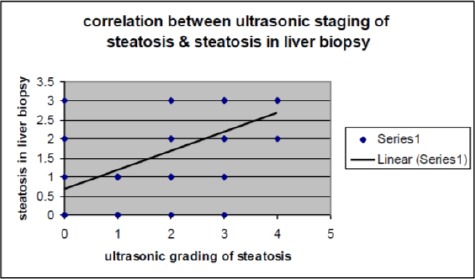
Correlation between ultrasonic staging of steatosis & steatosis in liver biopsy

We found that there was a significant correlation between both parameters. The sensitivity of sonar in detecting steatosis grade compared to biopsy was: 61% in grade 1, 25% in grade 2, and 75% in grade 3 (Figure 1).

**Figure 2:**
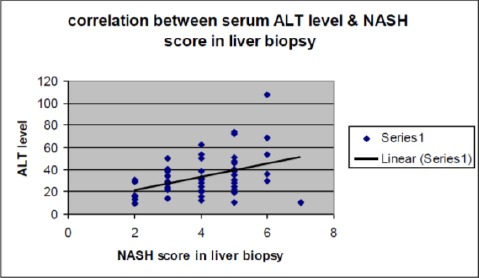
Correlation between serum alanine transferase (ALT) & non-alcoholic steatohepatitis (NASH) score in liver biopsy

Correlation study between steatosis in biopsy & other parameters revealed: 1-Highly significant positive correlation with ALT (Figure 2) (r = 0.8, P = 0.0001); 2- Positive correlation with AST (r = 0.2, P =0.05); 3- Positive correlation with GGT (R = 0.4, P = 0.05).

According to the results of the liver biopsy, patients were divided into 2 groups: Group 1included patients who have NASH (NASH and borderline NASH by biopsy). They were 34 patients out of the 54 patients. Group 2 includes patients who do not have NASH. They were 20 patients out of the 54 patients. Comparison between NASH & non-NASH groups as regards laboratory data revealed the significantly higher level of ALT, AST and GGT in NASH group than non- NASH group (P value < 0.001).

**Figure 3:**
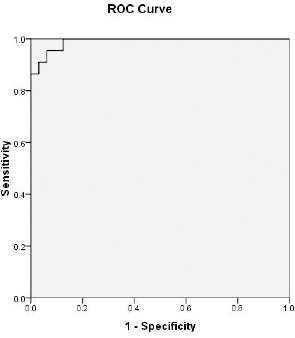
ROC curve to detect a cut off value of serum ALT that could predict the development of NASH

**Figure 4:**
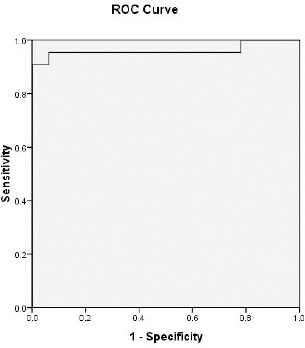
ROC curve to detect a cutoff value of serum aspartate transferase (AST) that could predict the development of NASH

**Figure 5:**
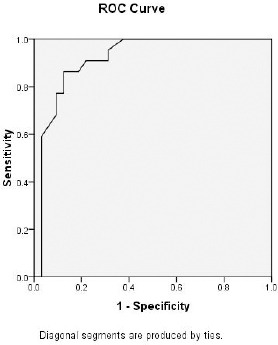
ROC curve to detect a cut off value of serum gamma-glutamyl-transferase (GGT) that could predict the development of NASH

Cutoff value for detection of NASH using ALT= 50.5 IU/L with sensitivity= 95.5% and specificity= 93.8% (Figure 3). Cutoff value for detection of NASH using AST= 56 IU/L with sensitivity= 90.5% and specificity= 100% (Figure 4). Cutoff value for detection of NASH using GGT= 60.5 IU/L with sensitivity= 86.4% and specificity= 87.5% (Figure 5).

## Discussion

NAFLD includes a spectrum of disorders defined essentially by macrovesicular hepatic fat infiltration occurring in people who do not consume alcohol in amounts considered harmful to the liver [9].

According to the results of the liver biopsy, patients were divided into 2 groups the first one included patients who were proven to have steatohepatitis and the second group included patients who do not have steatohepatitis. Although the present study cannot differentiate between both studied groups as regards sex, we cannot rely on this result because of the unequal distribution of sex in the study.

ALT and AST are important markers of liver injury. Their levels can be raised in many liver diseases. ALT is considered to be more accurate for hepatocyte injury and this is mainly attributed to its presence in high concentrations in hepatocyte cytosol. As regards AST, it has two types, cytosolic and mitochondrial and is found in the hepatocytes, cardiomyocytes, leukocytes and erythrocytes and other tissues [10].

In the present study, it was found that ALT, AST, and GGT were higher in patients with NASH in comparison to NAFLD patients, and the difference was highly significant. This agrees with Shi and colleagues, (2009) [10] who studied patients proven to have NAFLD by biopsy and found that ALT and AST levels of NASH group were higher than those of non-NASH group and found also that ALT is a reliable indicator of the severity of inflammation. Similarly, Fracanzani and colleagues, (2011) (studied patients with NAFLD and found that ALT levels were higher in steatohepatitis [11].

In contrast to this, Wong and colleagues. (2009) found that patients with ALT less than half the upper limit of normal may have the possibility of NASH [12]. Also, Oh and colleagues, (2006) found that raised ALT above normal values did not differentiate between patients who have steatohepatitis and patients who do not have steatohepatitis by liver biopsy [13].

As to AST, Ong and colleagues, (2005) found that AST was a reliable predictor of NASH [14]. The study done by Pulzi and colleagues, (2011) used a cutoff value of 30 IU/L for GGT that has a sensitivity 70% of and a specificity of 88.6% to distinguish NASH from non-NASH groups [15]. Tahan and colleagues, (2008) studied biopsy-proven NAFLD patients and divided them into normal and high GGT groups and found that there were no major differences regarding the degree of steatosis and inflammation [16].

As to ultrasonography, it is the most widely used and less invasive method used for NAFLD diagnosis. It can detect steatosis, which appears as hyperechogenic parenchyma, termed “bright liver”, and “blurring of the vascular margins” [17].

Regarding liver US, which was done to all patients with the same operator prior to biopsy, it was found that the prevalent steatotic grade in NASH group was grade 3 (37.5%), in comparison to the non-NASH group (most of them have grade 1 steatosis (75%). This may reflect that the degree of steatosis increases when patients develop steatohepatitis. The sensitivity of sonar in detecting steatosis compared to liver biopsy is 61% in grade 1, 25% of grade 2 and 75% in grade 3.

The results of the current study agree with the results of Fracanzani et al, (2011) who found that advanced steatosis independently predicts NASH [11]. Liver histology is the best diagnostic method for grading liver steatosis, inflammation and fibrosis, but it is not routinely used in patients with non-progressive fatty liver [18].

In the present study, the correlation between liver ultrasound and liver biopsy was highly significant in detecting the degree of hepatic steatosis and subsequently the development of NASH. Cutoff value for detection of NASH using ALT= 50.5 IU/L with sensitivity = 95.5% and specificity = 93.8%. In contrast to our results, Verma et al., 2013 found that there is no ideal ALT value that can predict NASH and advanced fibrosis [19]. The study was done by Somaye et al., 2011 found that using the cut-off value of 35 IU/L for serum ALT level, did not have a major contribution to stage NAFLD [20].

The present study showed that the cutoff value for detection of NASH using AST = 56 IU/L (international unit per litre) with sensitivity = 90.5% and specificity = 100%, and for GGT = 60.5 IU/L with sensitivity = 86.4% and specificity = 87.5%. In regard to this, Mikako and Hirofumi, 2012 stated that serum GGT value above 96.5 IU/L, predicted the advanced stage of fibrosis with 83% sensitivity and 69% specificity [21].

Although the present study tried to highlight the important role of US in NAFLD diagnosis and also the good correlation between US and biopsy results “if done by an experienced operator”, the quite novel role of this study is to elucidate also the promising role of liver enzymes as surrogate markers for NASH development. If we used these parameter levels, we can predict the possibility of NASH development and so careful follow-up of patients with a diet and lifestyle interventions to decrease the development of complications such as fibrosis, cirrhosis and hepatocellular carcinoma. This approach may avoid the need for a liver biopsy which may be refused by most patients and so we could miss patients who need lifestyle interventions and regular follow-up. The combination of all of the three parameters may suggest a higher possibility of NASH development and so more attention to patient follow-up. The importance of this study lies in the presentation of an alternative practical tool that could help in the diagnosis of NASH without the need for invasive tools such as liver biopsy.

It is worth to mention that there were some limitations in this study such as a discrepancy of a number of patients in both groups (NASH patients vs. non-NASH patients), and relatively small number of the studied patients (it was not easy to persuade patients to undergo liver biopsy).

In the present study, we concluded that liver enzymes level can be combined with abdominal sonar to predict the presence of NASH as a non-invasive method for diagnosis. Larger studies are needed to verify the level of liver enzymes that could predict accurately NASH development.
